# Crystal structure of 4-bromo­phenyl-2-oxo-2*H*-chromene-3-carboxyl­ate

**DOI:** 10.1107/S2056989015006738

**Published:** 2015-04-22

**Authors:** H. C. Devarajegowda, P. A. Suchetan, H. T. Srinivasa, S. Sreenivasa, B. S. Palakshamurthy

**Affiliations:** a Department of Physics, Yuvaraja’s College (Constituent College), University of Mysore, Mysore, Karnataka 570 005, India; b Department of Studies and Research in Chemistry, U.C.S., Tumkur University, Tumkur, Karnataka 572 103, India; c Raman Research Institute, C. V. Raman Avenue, Sadashivanagar, Bangalore, Karnataka 560080, India; d Department of Studies and Research in Chemistry, Tumkur University, Tumkur, Karnataka 572 103, India

**Keywords:** crystal structure, 2-oxo-2*H*-chromene, hydrogen bonding, π–π inter­actions

## Abstract

In the title compound, C_16_H_9_BrO_4_, the coumarin ring system is approximately planar, with an r.m.s deviation of the ten fitted non-H atoms of 0.031 Å, and forms a dihedral angle of 25.85 (10)° with the bromo­benzene ring. The carbonyl atoms are *syn*. In the crystal, mol­ecules are connected along [001] *via* C—H⋯O inter­actions, forming *C*(6) chains. Neighbouring *C*(6) chains are connected *via* several π–π inter­actions [range of centroid–centroid distances = 3.7254 (15)–3.7716 (16) Å], leading to sheets propagating in the *bc* plane.

## Related literature   

For related structures, see: Sreenivasa *et al.* (2013 ▸); Palakshamurthy, Sreenivasa *et al.* (2013 ▸); Palakshamurthy, Devarajegowda *et al.* (2013 ▸); Devarajegowda *et al.* (2013 ▸). For the biological activity and other applications of 2-oxo-2*H*-chromene derivatives, see: Abdel-Aziz *et al.* (2013 ▸); Kostova (2006 ▸); Chandrasekharan & Kelly (2002 ▸).
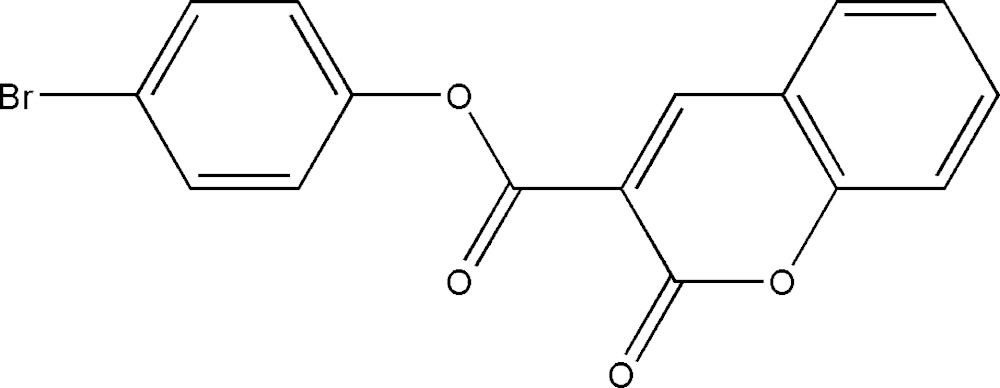


## Experimental   

### Crystal data  

C_16_H_9_BrO_4_
*M* *_r_* = 345.14
Monoclinic, 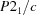

*a* = 16.0782 (10) Å
*b* = 7.2618 (4) Å
*c* = 12.7396 (8) Å
β = 113.311 (4)°
*V* = 1366.01 (15) Å^3^
*Z* = 4
Mo *K*α radiation
μ = 3.02 mm^−1^
*T* = 296 K
0.24 × 0.18 × 0.16 mm

### Data collection  

Bruker APEXII CCD diffractometer
Absorption correction: multi-scan (*SADABS*; Bruker, 2013 ▸) *T*_min_ = 0.526, *T*_max_ = 0.617
20483 measured reflections
2395 independent reflections
1831 reflections with *I* > 2σ(*I*)
*R*_int_ = 0.037

### Refinement  

*R*[*F* ^2^ > 2σ(*F* ^2^)] = 0.034
*wR*(*F* ^2^) = 0.079
*S* = 1.01
2395 reflections
191 parameters
H-atom parameters constrained
Δρ_max_ = 0.45 e Å^−3^
Δρ_min_ = −0.54 e Å^−3^

### 

Data collection: *APEX2* (Bruker, 2013 ▸); cell refinement: *SAINT* (Bruker, 2013 ▸); data reduction: *SAINT*; program(s) used to solve structure: *SHELXS97* (Sheldrick, 2008 ▸); program(s) used to refine structure: *SHELXL2014* (Sheldrick, 2015 ▸); molecular graphics: *Mercury* (Macrae *et al.*, 2008 ▸); software used to prepare material for publication: *SHELXL2014*.

## Supplementary Material

Crystal structure: contains datablock(s) I. DOI: 10.1107/S2056989015006738/tk5362sup1.cif

Structure factors: contains datablock(s) I. DOI: 10.1107/S2056989015006738/tk5362Isup2.hkl

Supporting information file. DOI: 10.1107/S2056989015006738/tk5362Isup3.cml

**Fig. 1.**
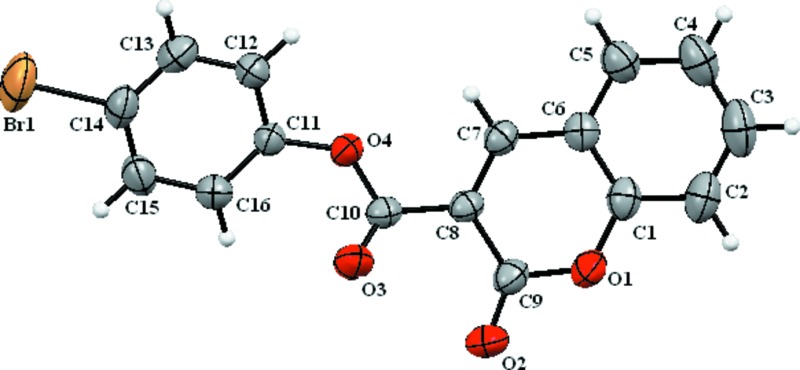
The molecular structure of the title compound, showing the atom labelling and displacement ellipsoids drawn at the 50% probability level.

**Fig. 2.**
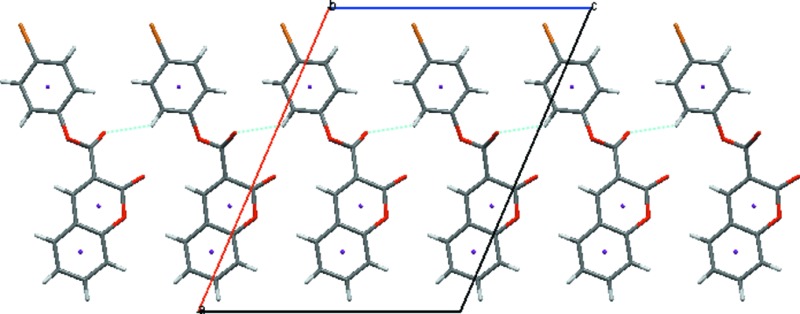
The crystal packing of the title compound via C—H···O interactions along [001]. Hydrogen bonds are shown as dashed lines.

. DOI: 10.1107/S2056989015006738/tk5362fig1.tif
The mol­ecular structure of the title compound, showing the atom labelling and displacement ellipsoids drawn at the 50% probability level.

via . DOI: 10.1107/S2056989015006738/tk5362fig2.tif
The crystal packing of the title compound *via* C—H⋯O inter­actions along [001]. Hydrogen bonds are shown as dashed lines.

**Fig. 3.**
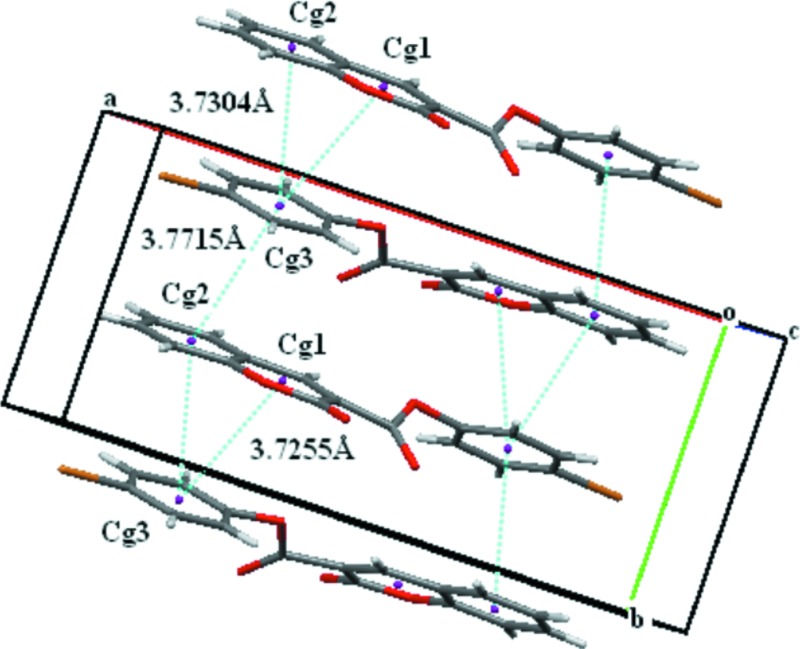
Various π–π interactions observed in the crystal packing

. DOI: 10.1107/S2056989015006738/tk5362fig3.tif
Various π–π inter­actions observed in the crystal packing

CCDC reference: 1057743

Additional supporting information:  crystallographic information; 3D view; checkCIF report

## Figures and Tables

**Table 1:** Hydrogen-bond geometry (, )

*D*H*A*	*D*H	H*A*	*D* *A*	*D*H*A*
C12H12O3^i^	0.93	2.40	3.124(3)	134

Symmetry code: (i) 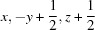.

## supplementary crystallographic information

### S1. Chemical context

Hetero cyclic compounds of 2-oxo-2*H*-chromenes display wide range of biological activities such as anti-HIV (Kostova, *et al.*, 2006), anti-cancer (Abdel-Aziz *et al.*,2013), etc. They also play a significant role as chemical sensors, fluorescent probes and laser dyes (Chandrasekharan *et al.*, 2002). In continuation of our work on 2-oxo-2*H*-chromene derivatives (Sreenivasa *et al.*, 2013; Palakshamurthy, Sreenivasa *et al.*, 2013; Palakshamurthy, Devarajegowda *et al.*, 2013; Devarajegowda, *et al.*,2013), in the present work we report the synthesis and crystal structure of 4-bromo­phenyl-2-oxo-2*H*-chromene-3-carboxyl­ate (I), an inter­mediate compound obtained during synthesis of coumarin–based Liquid Crystals (LCs).

### S2. Structural commentary

The dihedral angle between the coumarin ring and the bromo­benzene ring in (I) is 25.85 (10)°. Compared to this, the dihedral angle is 22.95 (11)° in 4'-cyano­biphenyl-4-yl-7-di­ethyl­amino-2-oxo-2*H*-chromene-3-carboxyl­ate (II) (Sreenivasa *et al.*, 2013), 62.97 (2)° in 4-(decyl­oxy)phenyl 2-oxo-7-tri­fluoro­methyl-2*H*-chromene-3-carboxyl­ate (III) (Palakshamurthy, Sreenivasa *et al.*, 2013), 21.00 (1)° in 4-(octyl­oxy)phenyl 2-oxo-2*H*-chromene-3-carboxyl­ate (IV) (Palakshamurthy, Devarajegowda *et al.*, 2013) and 54.46 (17)° in 4-[4-(heptyl­oxy)benzoyl­oxy] phenyl 2-oxo-7-tri­fluoro­methyl-2*H*-chromene-3-carboxyl­ate (V) (Devarajegowda, *et al.*, 2013). Further, in (I), the torsions C9—C8—C10—O3, O3—C10—O4—C11 and C12—C11—O4—C10 have values 27.6 (4), 6.3 (3) and 124.6 (2)°, respectively.

### S3. Supra­molecular features

In the crystal structure, the molecules are connected along [001] via C12—H12···O3 inter­actions forming C(6) chains (Fig 2., Table 2). Further, neighbouring C(6) chains are inter­locked via π···π inter­actions (Fig. 3), namely, Cg1··· Cg3^i^ [3.7254 (15) Å, i: 1-x, 1/2+y, 1/2-z] and Cg2··· Cg3^i,ii^ [3.7303 (16) and 3.7716 (16) Å, ii: 1-x, 1/2+y, 1/2-z], where Cg1, Cg2 and Cg3 are the centroids of the C6/C7/C8/C9/O1/C1, C1–C6 and C11–C16 rings, respectively). Overall, a two-dimensional architecture is observed in the *bc* plane.

### S4. Synthesis and crystallization

Coumarin 3-carb­oxy­lic acid (1.0 mmol), 4-bromo­phenol (1.0 mmol) and a catalytic amount of N,N-di­methyl­amino­pyrimidine (DMAP) were dissolved in anhydrous CH_2_Cl_2_. To this solution, a solution of di­cyclo­hexyl­carbodimide (DCC) in dried CH_2_Cl_2_ was added and stirred. After 24 h of stirring, di­cyclo­hexyl­urea was filtered off and the solution was concentrated. The solid residue obtained was purified by column chromatography on silica gel using CHCl_3_ as the eluent. Single crystals suitable for X-ray studies were grown by slow evaporation technique at room temperature using ethanol as the solvent.

### S5. Refinement

The H atoms were positioned with idealized geometry using a riding model with C—H = 0.93Å, and with 1.2*U*_eq_(C). Owing to poor agreement, several reflections, *i.e.* (0 2 5), (-1 0 2), (-2 0 8), (7 0 0) and (-7 2 5), were omitted from the final cycles of refinement.

### Crystal data

**Table:**

C_16_H_9_BrO_4_	Prism
*M**_r_* = 345.14	*D*_x_ = 1.678 Mg m^−^^3^
Monoclinic, *P*2_1_/*c*	Melting point: 523 K
Hall symbol: -P 2ybc	Mo *K*α radiation, λ = 0.71073 Å
*a* = 16.0782 (10) Å	Cell parameters from 2395 reflections
*b* = 7.2618 (4) Å	θ = 2.8–25.0°
*c* = 12.7396 (8) Å	µ = 3.02 mm^−^^1^
β = 113.311 (4)°	*T* = 296 K
*V* = 1366.01 (15) Å^3^	Prism, colourless
*Z* = 4	0.24 × 0.18 × 0.16 mm
*F*(000) = 688	

### Data collection

**Table:**

Bruker APEXII CCD diffractometer	2395 independent reflections
Radiation source: fine-focus sealed tube	1831 reflections with *I* > 2σ(*I*)
Graphite monochromator	*R*_int_ = 0.037
Detector resolution: 2.01 pixels mm^-1^	θ_max_ = 25.0°, θ_min_ = 2.8°
φ and ω scans	*h* = −19→19
Absorption correction: multi-scan (*SADABS*; Bruker, 2013)	*k* = −8→8
*T*_min_ = 0.526, *T*_max_ = 0.617	*l* = −15→15
20483 measured reflections	

### Refinement

**Table:**

Refinement on *F*^2^	Secondary atom site location: difference Fourier map
Least-squares matrix: full	Hydrogen site location: inferred from neighbouring sites
*R*[*F*^2^ > 2σ(*F*^2^)] = 0.034	H-atom parameters constrained
*wR*(*F*^2^) = 0.079	*w* = 1/[σ^2^(*F*_o_^2^) + (0.0319*P*)^2^ + 0.8274*P*] where *P* = (*F*_o_^2^ + 2*F*_c_^2^)/3
*S* = 1.01	(Δ/σ)_max_ = 0.001
2395 reflections	Δρ_max_ = 0.45 e Å^−^^3^
191 parameters	Δρ_min_ = −0.54 e Å^−^^3^
0 restraints	Extinction correction: *SHELXL2014* (Sheldrick, 2015), Fc^*^=kFc[1+0.001xFc^2^λ^3^/sin(2θ)]^-1/4^
0 constraints	Extinction coefficient: 0.0062 (6)
Primary atom site location: structure-invariant direct methods	

### Special details

**Table:**

Geometry. All e.s.d.'s (except the e.s.d. in the dihedral angle between two l.s. planes) are estimated using the full covariance matrix. The cell e.s.d.'s are taken into account individually in the estimation of e.s.d.'s in distances, angles and torsion angles; correlations between e.s.d.'s in cell parameters are only used when they are defined by crystal symmetry. An approximate (isotropic) treatment of cell e.s.d.'s is used for estimating e.s.d.'s involving l.s. planes.

### Fractional atomic coordinates and isotropic or equivalent isotropic displacement parameters (Å^2^)

**Table:**

	*x*	*y*	*z*	*U*_iso_*/*U*_eq_	
Br1	0.94359 (2)	0.14526 (6)	0.64830 (3)	0.0907 (2)	
O1	0.31144 (12)	0.1686 (2)	−0.06316 (14)	0.0517 (5)	
O2	0.44603 (13)	0.1964 (3)	−0.06745 (15)	0.0612 (5)	
O3	0.58965 (12)	0.2657 (3)	0.15291 (14)	0.0556 (5)	
O4	0.56890 (11)	0.0734 (2)	0.27929 (13)	0.0428 (4)	
C1	0.25543 (17)	0.1424 (3)	−0.0061 (2)	0.0460 (6)	
C2	0.1631 (2)	0.1504 (4)	−0.0694 (3)	0.0613 (8)	
H2	0.1403	0.1720	−0.1477	0.074*	
C3	0.1059 (2)	0.1257 (4)	−0.0142 (3)	0.0709 (9)	
H3	0.0436	0.1322	−0.0558	0.085*	
C4	0.1389 (2)	0.0915 (4)	0.1017 (3)	0.0673 (9)	
H4	0.0990	0.0734	0.1372	0.081*	
C5	0.23074 (18)	0.0840 (4)	0.1648 (3)	0.0547 (7)	
H5	0.2530	0.0613	0.2429	0.066*	
C6	0.29078 (17)	0.1108 (3)	0.1108 (2)	0.0413 (6)	
C7	0.38706 (16)	0.1145 (3)	0.1709 (2)	0.0394 (6)	
H7	0.4122	0.0939	0.2493	0.047*	
C8	0.44200 (16)	0.1472 (3)	0.11609 (19)	0.0364 (6)	
C9	0.40485 (18)	0.1736 (3)	−0.0079 (2)	0.0440 (6)	
C10	0.54071 (17)	0.1702 (3)	0.18007 (19)	0.0385 (6)	
C11	0.65769 (16)	0.0988 (3)	0.36007 (19)	0.0370 (5)	
C12	0.66608 (17)	0.1487 (3)	0.4677 (2)	0.0433 (6)	
H12	0.6149	0.1711	0.4827	0.052*	
C13	0.75178 (19)	0.1654 (4)	0.5536 (2)	0.0517 (7)	
H13	0.7590	0.2000	0.6270	0.062*	
C14	0.82608 (18)	0.1301 (4)	0.5288 (2)	0.0499 (7)	
C15	0.81735 (18)	0.0805 (4)	0.4214 (2)	0.0538 (7)	
H15	0.8686	0.0581	0.4065	0.065*	
C16	0.73210 (17)	0.0637 (4)	0.3352 (2)	0.0452 (6)	
H16	0.7251	0.0293	0.2619	0.054*	

### Atomic displacement parameters (Å^2^)

**Table:**

	*U*^11^	*U*^22^	*U*^33^	*U*^12^	*U*^13^	*U*^23^
Br1	0.0491 (2)	0.1081 (3)	0.0795 (3)	−0.01632 (19)	−0.01221 (17)	0.0173 (2)
O1	0.0460 (11)	0.0681 (12)	0.0343 (9)	0.0056 (9)	0.0087 (8)	−0.0015 (8)
O2	0.0622 (13)	0.0888 (15)	0.0368 (10)	0.0067 (11)	0.0240 (10)	0.0059 (10)
O3	0.0497 (11)	0.0724 (13)	0.0443 (10)	−0.0097 (10)	0.0181 (9)	0.0127 (9)
O4	0.0364 (9)	0.0547 (10)	0.0336 (9)	−0.0038 (8)	0.0100 (7)	0.0081 (8)
C1	0.0401 (15)	0.0421 (15)	0.0489 (15)	0.0008 (11)	0.0102 (13)	−0.0059 (12)
C2	0.0473 (18)	0.0615 (19)	0.0576 (18)	0.0004 (14)	0.0020 (15)	−0.0034 (14)
C3	0.0383 (17)	0.064 (2)	0.096 (3)	−0.0063 (14)	0.0115 (18)	−0.0058 (18)
C4	0.0465 (18)	0.064 (2)	0.094 (3)	−0.0078 (15)	0.0305 (18)	−0.0008 (18)
C5	0.0470 (17)	0.0564 (17)	0.0636 (17)	−0.0055 (14)	0.0250 (15)	0.0011 (14)
C6	0.0403 (14)	0.0363 (14)	0.0455 (14)	0.0000 (11)	0.0150 (12)	−0.0028 (11)
C7	0.0421 (14)	0.0375 (13)	0.0367 (13)	0.0018 (11)	0.0135 (11)	−0.0006 (10)
C8	0.0421 (14)	0.0352 (13)	0.0316 (12)	0.0027 (10)	0.0144 (11)	−0.0010 (10)
C9	0.0464 (15)	0.0485 (15)	0.0335 (13)	0.0059 (12)	0.0121 (12)	−0.0013 (11)
C10	0.0433 (14)	0.0409 (14)	0.0336 (13)	0.0007 (11)	0.0178 (11)	−0.0006 (10)
C11	0.0333 (13)	0.0405 (13)	0.0354 (12)	−0.0006 (11)	0.0117 (10)	0.0040 (10)
C12	0.0401 (15)	0.0506 (15)	0.0407 (14)	0.0037 (12)	0.0177 (12)	0.0031 (11)
C13	0.0576 (18)	0.0535 (16)	0.0374 (14)	−0.0025 (13)	0.0120 (13)	0.0006 (12)
C14	0.0381 (15)	0.0502 (16)	0.0487 (16)	−0.0054 (12)	0.0036 (12)	0.0084 (12)
C15	0.0375 (15)	0.0610 (17)	0.0630 (18)	0.0030 (13)	0.0200 (14)	0.0106 (14)
C16	0.0445 (15)	0.0535 (16)	0.0399 (13)	0.0011 (12)	0.0192 (12)	0.0023 (12)

### Geometric parameters (Å, º)

**Table:**

Br1—C14	1.903 (3)	C5—H5	0.9300
O1—C1	1.376 (3)	C6—C7	1.430 (3)
O1—C9	1.384 (3)	C7—C8	1.346 (3)
O2—C9	1.200 (3)	C7—H7	0.9300
O3—C10	1.198 (3)	C8—C9	1.463 (3)
O4—C10	1.358 (3)	C8—C10	1.479 (3)
O4—C11	1.403 (3)	C11—C12	1.372 (3)
C1—C2	1.382 (4)	C11—C16	1.377 (3)
C1—C6	1.387 (4)	C12—C13	1.385 (4)
C2—C3	1.373 (5)	C12—H12	0.9300
C2—H2	0.9300	C13—C14	1.375 (4)
C3—C4	1.380 (5)	C13—H13	0.9300
C3—H3	0.9300	C14—C15	1.368 (4)
C4—C5	1.374 (4)	C15—C16	1.381 (4)
C4—H4	0.9300	C15—H15	0.9300
C5—C6	1.402 (4)	C16—H16	0.9300
			
C1—O1—C9	122.67 (19)	C9—C8—C10	118.1 (2)
C10—O4—C11	118.88 (18)	O2—C9—O1	116.2 (2)
O1—C1—C2	117.5 (2)	O2—C9—C8	127.5 (2)
O1—C1—C6	121.0 (2)	O1—C9—C8	116.3 (2)
C2—C1—C6	121.5 (3)	O3—C10—O4	123.6 (2)
C3—C2—C1	118.6 (3)	O3—C10—C8	126.2 (2)
C3—C2—H2	120.7	O4—C10—C8	110.2 (2)
C1—C2—H2	120.7	C12—C11—C16	121.9 (2)
C2—C3—C4	121.3 (3)	C12—C11—O4	115.9 (2)
C2—C3—H3	119.4	C16—C11—O4	122.1 (2)
C4—C3—H3	119.4	C11—C12—C13	119.1 (2)
C5—C4—C3	120.2 (3)	C11—C12—H12	120.4
C5—C4—H4	119.9	C13—C12—H12	120.4
C3—C4—H4	119.9	C14—C13—C12	119.0 (2)
C4—C5—C6	119.8 (3)	C14—C13—H13	120.5
C4—C5—H5	120.1	C12—C13—H13	120.5
C6—C5—H5	120.1	C15—C14—C13	121.6 (2)
C1—C6—C5	118.7 (2)	C15—C14—Br1	119.5 (2)
C1—C6—C7	117.9 (2)	C13—C14—Br1	118.9 (2)
C5—C6—C7	123.3 (2)	C14—C15—C16	119.7 (2)
C8—C7—C6	121.3 (2)	C14—C15—H15	120.2
C8—C7—H7	119.4	C16—C15—H15	120.2
C6—C7—H7	119.4	C11—C16—C15	118.7 (2)
C7—C8—C9	120.8 (2)	C11—C16—H16	120.7
C7—C8—C10	121.0 (2)	C15—C16—H16	120.7
			
C9—O1—C1—C2	176.4 (2)	C7—C8—C9—O1	2.0 (3)
C9—O1—C1—C6	−3.1 (4)	C10—C8—C9—O1	−173.9 (2)
O1—C1—C2—C3	−179.7 (2)	C11—O4—C10—O3	6.3 (3)
C6—C1—C2—C3	−0.2 (4)	C11—O4—C10—C8	−170.71 (19)
C1—C2—C3—C4	−0.7 (4)	C7—C8—C10—O3	−148.3 (3)
C2—C3—C4—C5	0.9 (5)	C9—C8—C10—O3	27.6 (4)
C3—C4—C5—C6	−0.2 (4)	C7—C8—C10—O4	28.7 (3)
O1—C1—C6—C5	−179.7 (2)	C9—C8—C10—O4	−155.5 (2)
C2—C1—C6—C5	0.9 (4)	C10—O4—C11—C12	124.6 (2)
O1—C1—C6—C7	2.8 (3)	C10—O4—C11—C16	−59.7 (3)
C2—C1—C6—C7	−176.7 (2)	C16—C11—C12—C13	0.4 (4)
C4—C5—C6—C1	−0.7 (4)	O4—C11—C12—C13	176.2 (2)
C4—C5—C6—C7	176.7 (3)	C11—C12—C13—C14	−0.5 (4)
C1—C6—C7—C8	−0.1 (3)	C12—C13—C14—C15	0.5 (4)
C5—C6—C7—C8	−177.6 (2)	C12—C13—C14—Br1	−177.94 (19)
C6—C7—C8—C9	−2.2 (3)	C13—C14—C15—C16	−0.5 (4)
C6—C7—C8—C10	173.5 (2)	Br1—C14—C15—C16	178.0 (2)
C1—O1—C9—O2	179.7 (2)	C12—C11—C16—C15	−0.3 (4)
C1—O1—C9—C8	0.7 (3)	O4—C11—C16—C15	−175.9 (2)
C7—C8—C9—O2	−176.9 (3)	C14—C15—C16—C11	0.4 (4)
C10—C8—C9—O2	7.3 (4)		

### Hydrogen-bond geometry (Å, º)

**Table:**

*D*—H···*A*	*D*—H	H···*A*	*D*···*A*	*D*—H···*A*
C12—H12···O3^i^	0.93	2.40	3.124 (3)	134

Symmetry code: (i) *x*, −*y*+1/2, *z*+1/2.
