# Supplementary figures and images for: Crystal structure of 4-bromo­phenyl-2-oxo-2H-chromene-3-carboxyl­ate
Source: Acta Crystallogr E Crystallogr Commun. 2015 Apr 22;71(Pt 5):o326–7. doi: 10.1107/S2056989015006738 (PMC4420041; doi:10.1107/S2056989015006738)

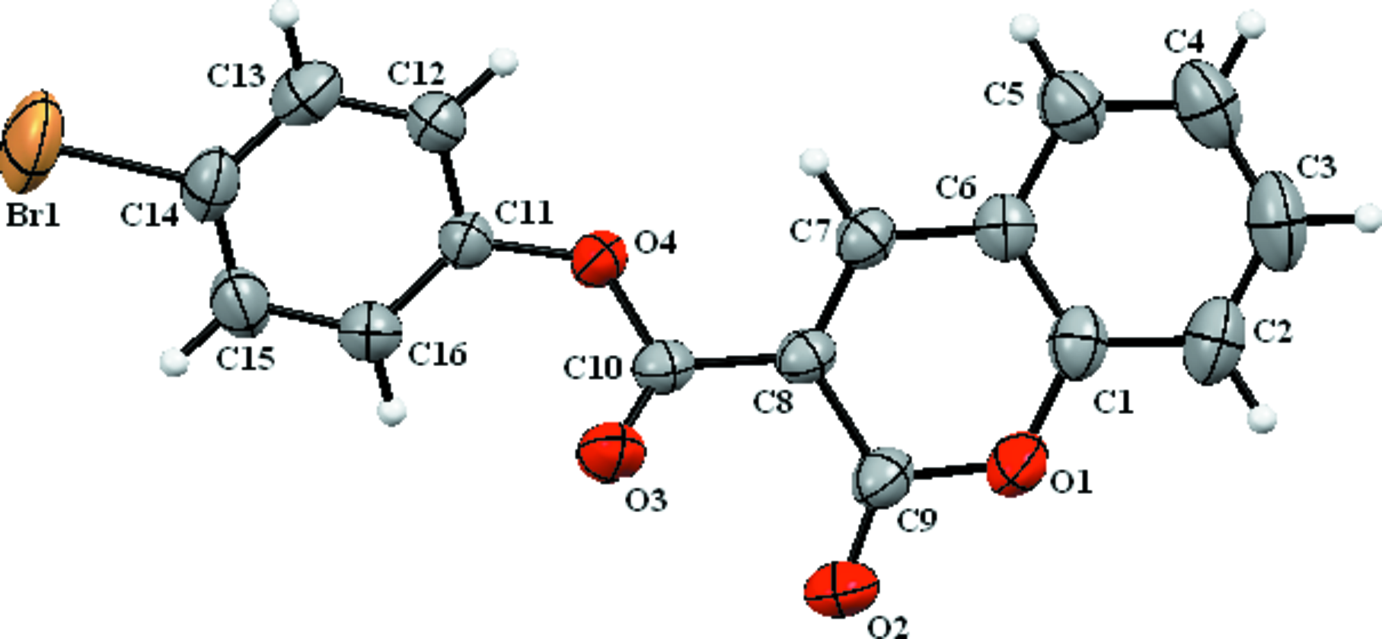

Supplement: Supplementary file 4 [file e-71-0o326-fig1.tif]

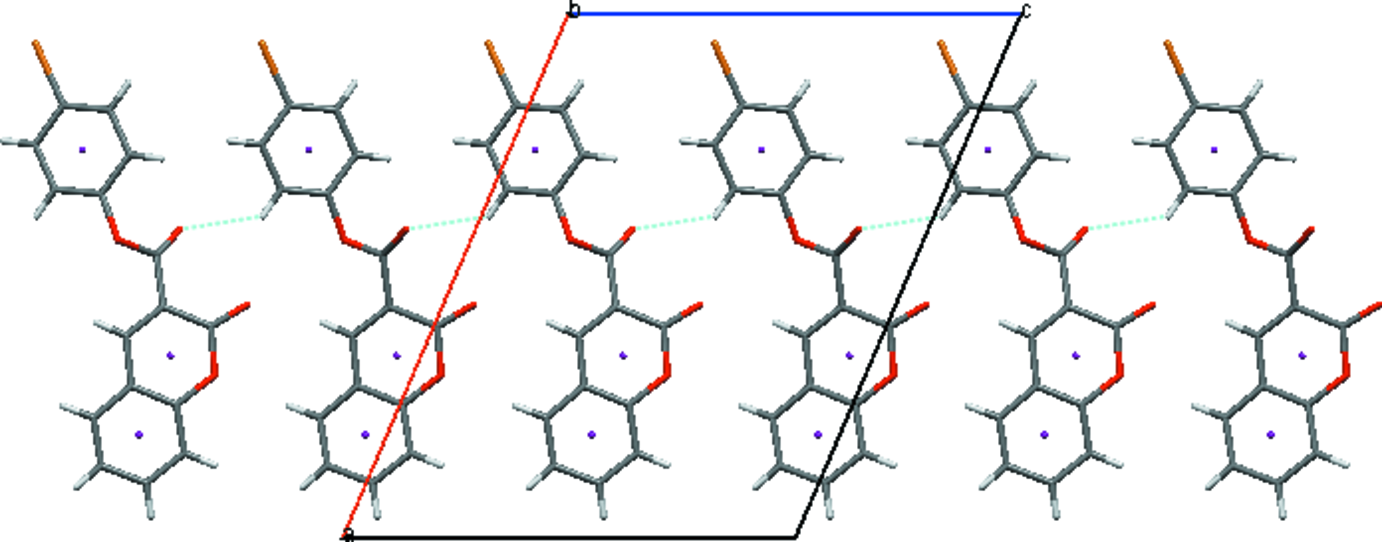

Supplement: Supplementary file 5 [file e-71-0o326-fig2.tif]

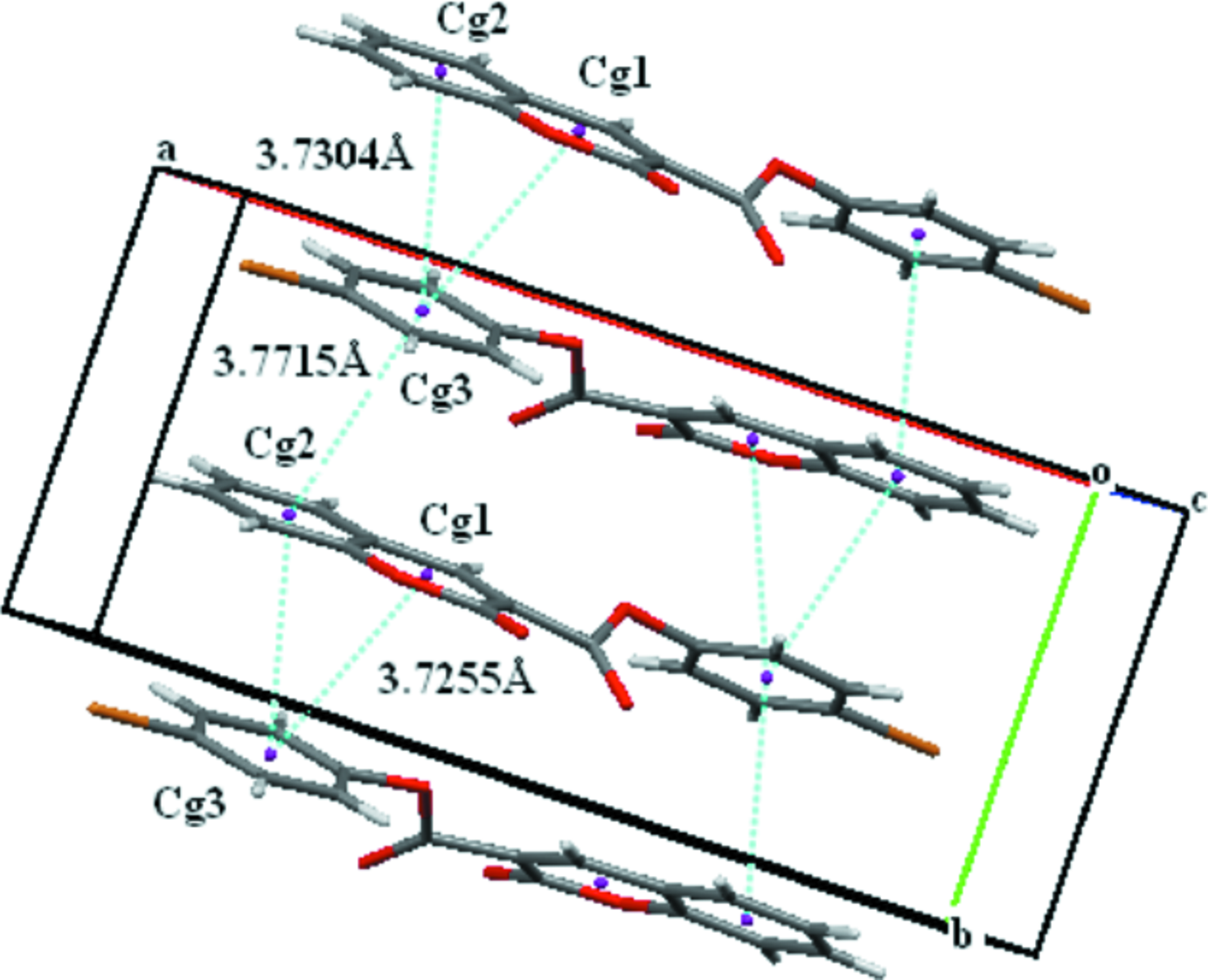

Supplement: Supplementary file 6 [file e-71-0o326-fig3.tif]
